# The Case of "Dr." Collins

**Published:** 1898-07-09

**Authors:** 


### The Hospital

A Journal of

The Medical Sciences and Hospital Administration.

Vol. XXIV.—No. 615.] Edited by Sir Henry Burdett, K.C.B., and Solomon C. Smith, M.D., M.R.C.P. [July 9, 1898.

### The Case of "Dr." Collins.

The conviction of Dr. Collins for causing the death of Mrs. Uzielli by an "illegal operation," or, in other words, by procuring abortion, may well serve to call attention to one of the gravest social scandals of the close of the century. It has long been notorious, as a matter of common talk and knowledge, that many married women of the idle and wealthy classes have so far lost not only the maternal instinct, but also the sense of duty by which that instinct might be replaced, as to shrink from the cares and responsibilities, or, in plain English, from the trouble of bearing and rearing children. Among the industrial classes also, if we may judge from columns of advertisements which appear in certain inferior newspapers, the same feeling has been gaining ground; and the jury which convicted Collins had ample justification for the rider which they appended to their verdict, and in which they expressed their "deep concern at and condemnation of the growing tendency on the part of a certain section of the community who, as proved by the evidence in this case, avail themselves of their marital rights, but try to evade the responsibility arising therefrom." The homely language of the Liturgy, which warns those who use it against entering into the state of matrimony "like brute beasts that have no understanding," might have been still more appropriate, were it not that these cherish and care for their offspring, while the class of women against whom the presentment of the jury was directed fall far short even of the purely animal standard. As long as women of fashion and station set the example of avoiding childbearing, and as long as the wives of the comparatively poor are content to follow it, there will be no lack of persons who will offer to provide remedies for "female irregularities," and who will be prepared, after a sufficient amount of money has been extorted from their clients in exchange for inert drugs, to proceed to operative measures if they can obtain sufficient payment. The present state of the law, which holds the operator and the woman to be equally guilty, is logically unassailable; but, as it practically prevents either from giving information against the other, it seems calculated rather to defeat than to further the ends of justice. These two persons would usually be the only ones who would possess complete information as to the facts; and circumstantial evidence, in such cases, would usually be wanting, and would seldom be trustworthy. Two qualified medical men, Ady and Collins, have been sentenced within the last few months, both of them having previously been removed from the Medical Register by the Council; but no one who is at all conversant with the conditions of modern life can for a moment doubt that those who have been punished have borne but a small proportion to those who have escaped.

We have heard that some women endeavour to salve their consciences by the old theological belief that the unborn child receives its soul at the time of quickening, and that, prior to this time, it need not be regarded as a living existence. It is needless to say that such a belief derives no support from physiology; and that, from the moment of conception, the unborn child is a potential human being, with as much right to the enjoyment of life, whether in this world or in the world to come, as any of its progenitors. In other words, artificial abortion is child murder, pure and simple; and none of the excuses which have been framed for it will bear a single moment's consideration. The women who submit to treatment which is intended to produce abortion are themselves accomplices in child murder; and it is the duty of all who are concerned in the development of public opinion to press this truth home, and to show the worthlessness of the flimsy excuses which are put forward in defence or in extenuation of the practice. The case is one in which the Press and the Pulpit should speak with no uncertain sound, and in which the powerful influence of legitimate leaders of Society might be exerted with the greatest possible advantage. Not the least of the evils arising from the feeling which now, in too many quarters prevails, is that it tends to the encouragement of immorality among unmarried women, who may easily be led to fancy, from what they hear around them, that the consequences of sexual misconduct need not be very serious after all, and that they may sin without risk of shame or disgrace. Such a belief, it is hardly necessary to say, tends to lower the moral standard of all the women among whom it prevails, and to render them less fitted to be the friends and companions of their husbands, or the guides and protectors of their children. Fortunately, events of this kind are calculated to provoke reaction; and we believe that the honesty and the sound sense of our countrywomen will before long be enlisted in antagonism to the prevalence of a custom which, derived originally from foreign sources, has increased among us until it has assumed formidable dimensions. We cannot believe that the sanctity of the English hearth and home will continue to be polluted by the practices to which we have referred, and which the events of the last few weeks have forced into such painful notoriety.

